# DNA photo-cross-linking using a pyranocarbazole-modified oligodeoxynucleotide with a d-threoninol linker†

**DOI:** 10.1039/c9ra06145b

**Published:** 2019-09-27

**Authors:** Kenzo Fujimoto, Tsubasa Yamaguchi, Takahiro Inatsugi, Masahiko Takamura, Isao Ishimaru, Ayako Koto, Shigetaka Nakamura

**Affiliations:** Department of Advanced Institute Science and Technology, Japan Advanced Institute of Science and Technology Asahidai 1-1 Nomi Ishikawa 923-1292 Japan kenzo@jaist.ac.jp; Advanced Materials Research Laboratory, Advanced Technology Research Department, In statute of Surface Science and Technology, NICCA CHEMICAL CO., LTD. 23-1, 4-Chome, Bunkyo Fukui-City 910-8670 Japan

## Abstract

An alternative photo-cross-linker having a d-threoninol skeleton instead of the 2′-deoxyribose backbone in 3-cyanovinylcarbazole (^CNV^K) was investigated to improve the photoreactivity of photo-cross-linkers; the photo-cross-linking rate of 3-cyanovinylcarbazole with d-threoninol (^CNV^D) was found to be greater than that of ^CNV^K. Therefore, in this study, a novel photo-cross-linker having pyranocarbazole (^PC^X) and d-threoninol instead of the 2′-deoxyribose backbone in ^PC^X (^PCX^D) was developed. The ^PCX^D in double-stranded DNA photo-cross-linked to a pyrimidine base at the −1 position of a complementary strand similar to ^PC^X. Furthermore, the photoreactivity of ^PCX^D was significantly higher than that of ^PC^X. The introduction of d-threoninol improved the reactivity of pyranocarbazole to cytosine, the use of ^PCX^D may extend the applicability of the photo-cross-linking reaction for DNA manipulation. In particular, this novel photo-cross-linker can contribute to the photochemical regulation of gene expression or biological events in a living cell.

## Abstract 2

An alternative more efficient photo-cross-linker having a d-threoninol skeleton instead of the 2′-deoxyribose backbone in pyranocarbazole was investigated to improve the photoreactivity of photo-cross-linkers.

## Graphical abstract

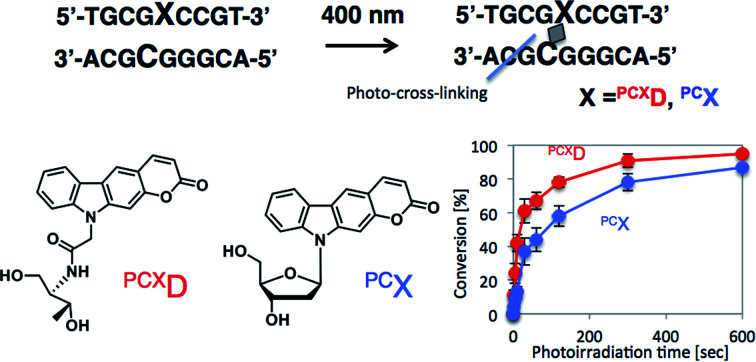

## Introduction

**Fig. 1:**
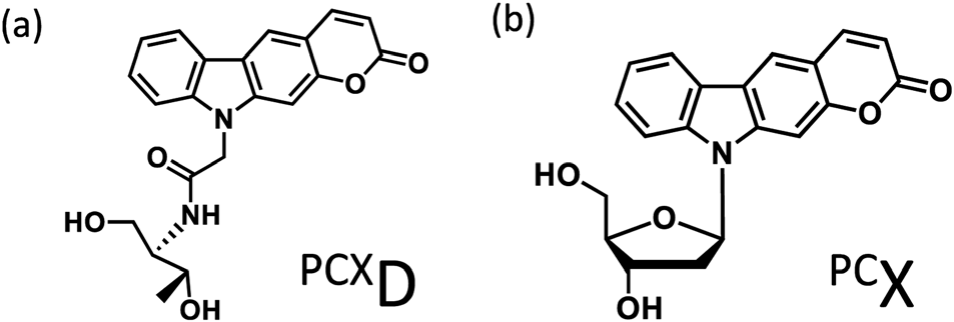
Ultrafast photo-cross-linker (a) pyranocarbazole with d-threoninol (^PCX^D); (b) pyranocarbazole (^PC^X).

Photo-cross-linking reactions between biomolecules are used for various applications such as screening antigen interactions^1^ and improving detection sensitivity^2^ and the stability of biomolecular complexes.^3^ In particular, the use of DNA photo-cross-linking in the formation of a thymine dimer induced by UV-irradiation^4^ and interstrand photo-cross-linking with psoralen^5^ has been reported. In recent years, with the development of nucleic acid medicine and DNA nanotechnology, these have been used for the photochemical regulation of an antisense effect,^6^ improvement in stability of a DNA nanostructure,^7^ and other applications. Many photo-cross-linkers such as bromouracil^8^ and benzophenone^9^ have also been reported. Among these, DNA photo-cross-linking *via* [2 + 2] photocycloaddition by psoralens^5^ and coumarins^10^ is useful for detection, manipulation, and regulation of nucleic acids because photoreversible manipulation is possible. Compared to the enzymatic method, photo-cross-linking can be used under a wide range of conditions without the addition of reagents. Although the intracellular usage of photo-cross-linking has already been reported, these require UV-irradiation, which pose a limitation for use in living cells because of phototoxicity. We report pyranocarbazole (^PC^X) as a photo-cross-linker that can photo-cross-link to pyrimidine in complementary DNA or RNA strand under visible light.^11^ It was anticipated that his photo-cross-linker would accelerate the intracellular application of nucleic acid photo-cross-linking such as photochemical regulation of gene expression^12^ and detection of RNA strand;^13^ however, photo-cross-linking using ^PC^X to cytosine requires photoirradiation for 1 min, and it is necessary to speed up this process. Besides, the ribose backbone, d-threoninol backbone,^14^ and serinol backbone^15^ have been reported as the backbone of the artificial nucleic acid. It was determined that 3-cyanovinylcarbazole modified d-threoninol (^CNV^D)^16^ considerably accelerated the photo-cross-linking reaction with cytosine using the d-threoninol backbone (^PCX^D) (Fig. 1).

## Results and discussion

**Fig. 2:**
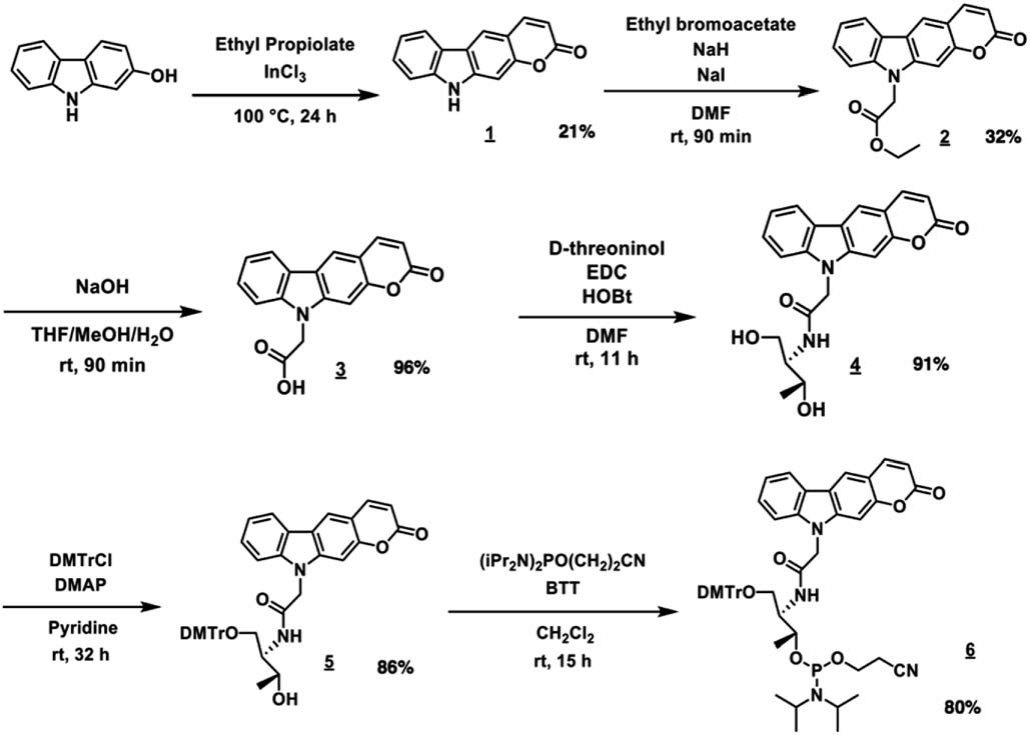
Synthesis of ^PCX^D phosphoramidite.

^PCX^D was successfully synthesized according to the reaction scheme shown in Fig. 2. The ^PCX^D was phosphoramidited using a general method^17^ after 5′ DMTr protection to synthesize the oligodeoxynucleotide (ODN) containing ^PCX^D. The 9 mer ODN which has A, T, G, C having deoxyribose and ^PCX^D having d-threoninol was synthesized with an automated DNA synthesis machine, and then, it was deprotected using 28% ammonium solution with a general method. After HPLC purification, it was analyzed using Matrix assisted Laser Desorption/Ionization (MALDI) analysis (Table 1).

First, we demonstrated the photo-cross-linking of ^PCX^D in double-stranded DNA. The duplex containing ^PCX^D was irradiated with 400 nm and analyzed using ultrahigh-performance liquid chromatography (UPLC). In the case of the pyrimidine base at the −1 position on the complementary ODN (cODN) strand, new peak identical to the photodimer appeared after photoirradiation, although such new peak did not appear in the case of the purine base at the −1 position on the cODN strand. The new peak was identified to photoadduct of PCXD-ODN(C) and its complementary strand (found = 5591.2, calcd [M + H]^+^ = 5591.0) by MALDI-TOF-MS analysis. This suggests that the photo-cross-linking reaction occurred only in the case of the pyrimidine base at the −1 position on the cODN strand.

**Table 1:** Sequence of ODN

Entry	Sequence (5′–3′)	Calculated [M + H]^+^	Found
^PCX^D-ODN(C)	TGCG^PCX^DCCGT	2843.53	2843.60
^PC^X-ODN(C)	TGCG^PC^XCCGT	2823.31	2823.81
^PCX^D-ODN(T)	TGCA^PCX^DCCGT	2827.84	2828.94
^PC^X-ODN(T)	TGCA^PC^XCCGT	2807.52	2807.43

**Fig. 3:**
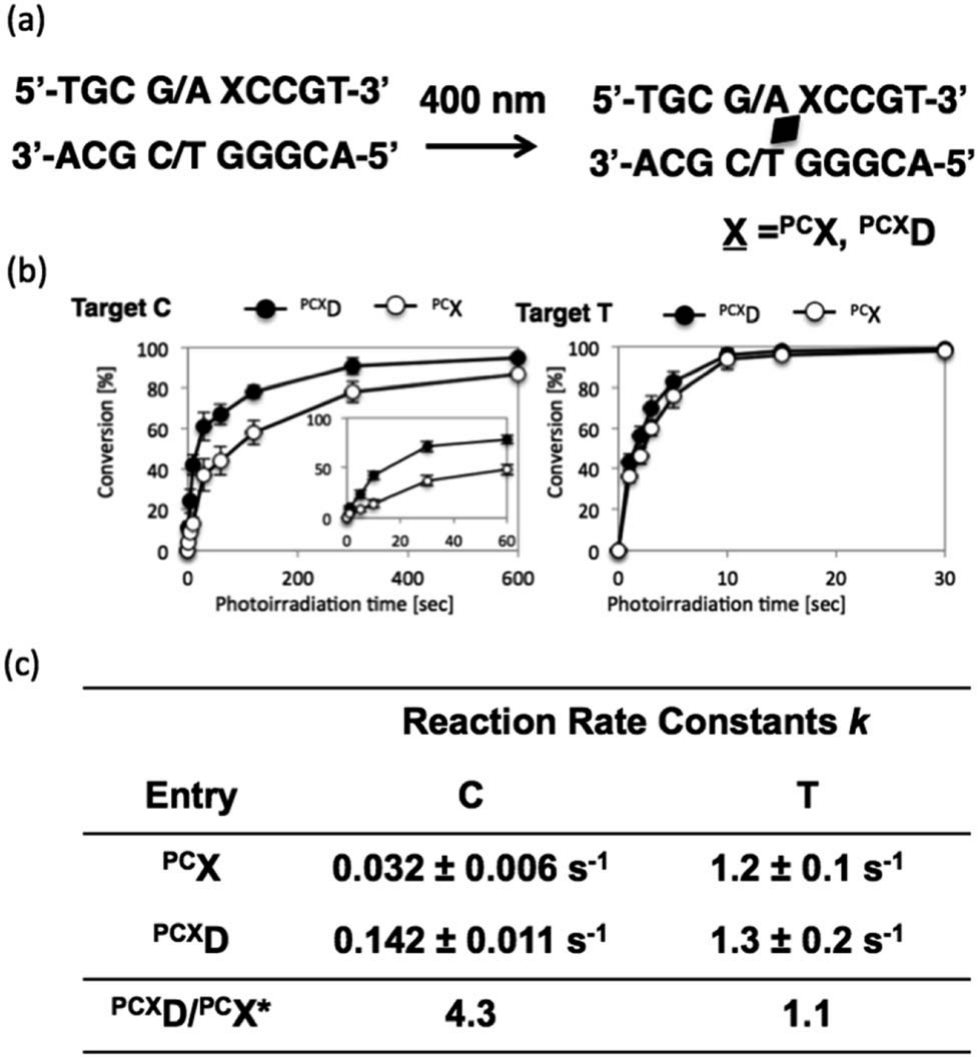
Photo-cross-inking of ^PCX^D oligonucleotide in dsDNA. (a) Schematic of photo-cross-linking; (b) time course of photo-cross-linking to cytosine and thymine; inserted figure is an enlarged figure from 0 s to 60 s. (c) Summarized reaction rate.

To analyze the photoreactivity of ^PCX^D, the time course of the photoreaction was monitored, and then, the reaction rate constant was analyzed with the assumption of first-order reaction kinetics. As shown in Fig. 3, the reaction rate constant of ^PCX^D with cytosine is 4.3-fold larger than that of ^PC^X, suggesting that the relatively flexible d-threoninol skeleton elevates the accessibility of the reactive double bond to the cytosine base on the complementary strand. The same effect was observed in the case of thymine as the target base of ^PCX^D; the reaction rate constant was 1.1-folds larger than that of ^PC^X. The difference in the reactivity between the thymine and the cytosine bases decreased and the photoreactivity enhanced compared with ^PC^X, which indicates that the use of ^PCX^D extends the applicability of the photo-cross-linking reaction for DNA manipulation.

To evaluate the duplex stability of ODN(^PCX^D), the melting temperature (*T*_m_) of the duplex consisting of ODN(A^PCX^D) and ODN(GT) was evaluated and compared with the duplex including ^PC^X. The sigmoidal melting curve of ODN(^PCX^D)/ODN(T) duplex was observed, which suggests ODN(^PCX^D) and cODN(GT) formed a duplex structure. Then, we calculated the thermodynamic parameter from van't Hoff plots.^18^ The Δ*S* through ODN(^PCX^D)/ODN(T) hybridization was smaller than that of ODN(^PC^X)/ODN(T), suggesting that a relatively flexibility structure of ^PCX^D inhibited the entropic loss *via* hybridization (Table 2). Therefore, ^PCX^D has a higher flexibility than ^PC^X, and it induces a higher photo-cross-linking ability in cytosine than ^PC^X. In addition, ^PC^X and ^PCX^D containing pyranocarbazole moieties have lower *T*_m_ values ​​compared to ^CNV^K and ^CNV^D containing 3-cyanovinylcarbazole moiety. When the polarity of pyranocarbazole (log *P* = 1.12) and 3-cyanovinylcarbazole (log *P* = 1.35) was examined,^19^ it was found that pyranocarbazole has a smaller log *P* and is hydrophilic. Therefore, it is considered that the *T*_m_ value of the duplex containing 3-cyanovinylcarbazole is high and the entropy loss is large because of the stacking between bases and hydrophobic interactions.

**Table 2:** Thermodynamic parameter of each photo-cross-linker

Entry	*T*_m_ [°C]	ΔΔ*G*_37_ (kcal mol^−1^)	Δ*H* (kcal mol^−1^)	Δ*S* (cal mol^−1^ K^−1^)
^PCX^D	26	−7.4	−16.7	−30.0
^PC^X	33	−0.58	−21.6	−67.7
^CNV^D	29	−7.3	−33.8	−85.6
^CNV^K	34	−7.5	−63.6	−181.1

**Fig. 4:**
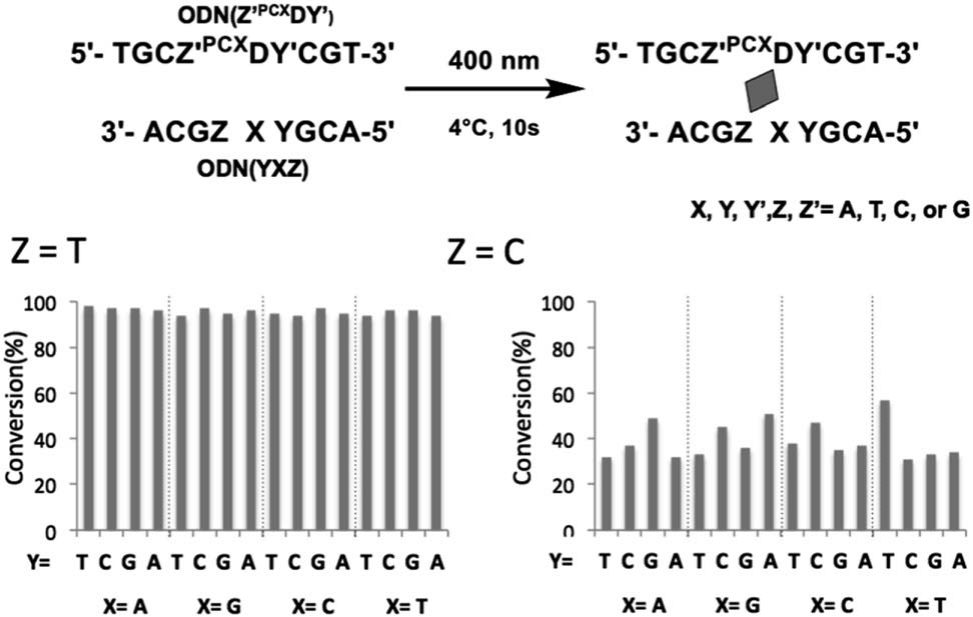
Effect of surrounding base of ^PCX^D on its photoreactivity in dsDNA. 10 μM DNA in 50 mM cacodylate buffer (pH 7.4) containing 100 mM NaCl was 400 nm-irradiated for 10 s at 4 °C.

To evaluate the effects of the surrounding bases on the photo-cross-linking of ODNs containing ^PCX^D, double strand with all variations of base pairs at the −1, +1 position of ^PCX^D and four different bases on the counter position to ^PCX^D in cODN were prepared; the conversion after 10 s of 400 nm irradiation was evaluated by UPLC. As shown in Fig. 4, in the case of T at the *Z* position in cODN, over 90% of the photo-cross-linking was obtained with 400 nm irradiation for 10 s. In the case of C at the *Z* position, photo-cross-linking was observed. However, in the case of A or G at the *Z* position, photo-cross-linking was not observed. These results indicate that the photo-cross-linking of ^PCX^D has similar pyrimidine selectivity as those of ^CNV^K, ^CNV^D, and ^PC^X. In addition, some differences in the reactivity were not observed among these duplexes, which indicated that the mechanism of effects such as local duplex stability and local electrostatic environment around the pyranocarbazole moiety remained does not affect the photo-cross-linking.

## Experimental

### General

^1^H NMR spectra were measured on a Bruker AVANCE III 400 system. Mass spectra were recorded on a Voyager PRO-SF, Applied Biosystems and HRMS were measured on a Solarix-JA (Bruker) HPLC was performed on a Chemcosorb 5-ODS-H column with JASCO PU-980, HG-980-31, DG-980-50 system equipped with a JASCO UV 970 detector at 260 nm. Reagents for synthesis for compound 1 to 5 were purchase from Aldrich, Wako, and TCI. Phosphoramidites of ^PCX^D were synthesized by the procedure as follows. Other phosphoramidite reagents for the DNA sysnthesizer such as A, G, C, T-β-cyanoethyl phosphoramidite, and CPG support were purchased form Glen research. Other oligonucleotides were purchased from Fasmac (Japan) and used without farther purification. The synthesized ODN having deoxyribose backbone except ^PCX^D.

### Synthetic procedures

#### Compound 2

Pyranocarbazole (1) (628 mg, 2.67 mmol) and NaH (60% oil suspension, 320 mg, 13.4 mmol) were solved in dry DMF (10 mL) and stirred for 20 min on ice under N_2_ atmosphere. Ethyl bromoacetate (0.59 mL, 5.34 mmol) and NaI (1.2 g, 8.0 mmol) was drop-wisely added over 30 min and stirred for 90 min at room temperature. Reaction mixture was poured onto water (200 mL) and extracted with chloroform and then dried over sodium sulfate. After removal of the solvent the residue was subjected to silica gel column chromatography (CHCl_3_) to afford 2 (yellow solid, 1.4 g, 4.6 mmol, 32%). ^1^H NMR (400 MHz, CDCl_3_) 8.06 (s, 1H), 8.01 (d, 1H, *J* = 7.6 Hz), 7.8 (d, 1H, *J* = 8.0 Hz), 7.44 (m, 1H), 7.25 (d, 1H, *J* = 9.2 Hz), 7.24 (m, 1H), 7.13 (s, 1H), 6.26 (d, 1H, *J* = 9.2 Hz), 4.91 (s, 2H), 4.17 (m, 1H), 1.19 (t, 3H, *J* = 7.2 Hz). ^13^C NMR (100 MHz, CDCl_3_) 167.8, 161.6, 153.5, 144.6, 143.1, 141.7, 126.9, 122.7, 121.1, 121.0, 120.6, 119.7, 112.9, 112.7, 108.9, 96.1, 62.2, 45.0, 14.3. ESI-FT-ICR MS(HRMS) calcd for [M + H]^+^: 322.1073, found: 322.1073.

#### Compound 3

2 (402 mg, 1.25 mmol) and NaOH (110 mg, 2.75 mmol) were solved in THF/MeOH/H_2_O (3 : 2 : 1, 18 mL) and stirred for 90 min at room temperature. After the addition of 1 N HCl (150 mL), reaction mixture was extracted with EtOAc (150 mL) and washed with 1 N HCl. Organic layer was dried over MgSO_4_ and evaporated to afford 3 (yellow solid, 1.2 g, 4.4 mmol, 96%). ^1^H NMR (400 MHz, DMSO-d_6_) 13.17 (s, 1H), 8.50 (s, 1H), 8.19 (d, 2H, *J* = 9.2 Hz), 7.65 (s, 1H), 7.59 (d, 1H, *J* = 8.4 Hz), 7.50 (m, 1H), 7.30 (t, 1H, *J* = 6.8 Hz), 6.36 (m, 1H), 5.30 (d, 2H). ^13^C NMR (100 MHz, DMSO-d_6_) 169.8, 160.6, 152.8, 145.4, 143.0, 141.6, 126.5, 121.9, 120.4, 120.3, 120.1, 120.0, 112.0, 111.9, 109.7, 96.4, 44.2. ESI-FT-ICR MS(HRMS) calcd for [M + H]^+^: 294.0760, found: 294.0762.

#### Compound 4

3 (156 mg, 0.53 mmol) and d-threoninol (111 mg, 1.06 mmol) were added to dry DMF (10 mL) containing 1-ethyl-3-(3-dimethylaminopropyl)carbodiimide hydrochloride (203 mg, 1.06 mmol) and 1-hydroxybenzotriazole (143 mg, 1.06 mmol), and stirred for 11 h at room temperature. NaCl aq. soln was added and obtained precipitate was collected and dried under vacuum to afford 4 (yellow solid, 1.5 g, 4.1 mmol, 91%). ^1^H NMR (400 MHz, DMSO-d_6_) 8.50 (s, 1H), 8.18–8.21 (m, 2H), 8.00 (d, 1H, *J* = 8.8 Hz), 7.58–7.60 (m, 2H), 7.50 (t, 1H, *J* = 7.2 Hz), 7.30 (t, 1H, *J* = 7.2 Hz), 6.34 (d, 1H, *J* = 9.2 Hz), 5.17 (d, 2H, *J* = 3.6 Hz), 4.72 (d, 2H, *J* = 4.2 Hz), 4.64 (t, 3H, *J* = 4.2 Hz), 3.91 (m, 1H), 3.65 (br, 1H), 3.47–3.54 (m, 1H), 3.36–3.41 (m, 1H), 1.02 (d, 3H, *J* = 6.4 Hz). ^13^C NMR (100 MHz, DMSO-d_6_) 167.0, 160.6, 152.8, 145.4, 143.1, 141.8, 126.4, 121.8, 120.3, 120.1, 120.1, 119.9, 111.9, 111.8, 109.8, 96.3, 63.9, 60.5, 55.9, 45.6, 20.3. ESI-FT-ICR MS(HRMS) calcd for [M + H]^+^: 381.1444, found: 381.1445. The molar extinction coefficient of ^PCX^D is 4400 M^−1^ cm^−1^.

#### Compound 5

4 (97.6 mg, 0.26 mmol), 4,4′-dimethoxytritylchloride (105 mg, 0.31 mmol) and 4-dimethyl-aminopyridine (105 mg, 0.31 mmol) in dry pyridine (1 mL) was stirred at room temperature for 18 h. The reaction mixture was diluted with CHCl_3_, washed with H_2_O and organic layer was dried over NaSO_4_. After the removal of the solvent, residue was subjected to silica gel column chromatography (CHCl_3_ with 0.1% TEA) to afford 5 (yellow solid, 0.63 g, 86%). ^1^H NMR (400 MHz, DMSO-d_6_) 8.47 (s, 1H) 8.17 (t, 3H, *J* = 8.8 Hz) 7.59 (d, 1H, *J* = 8.4 Hz), 7.57 (s, 1H), 7.44 (t, 1H, *J* = 8.8 Hz) 7.33–7.18 (m, 10H), 6.78 (t, 4H, *J* = 8.8 Hz), 6.31 (d, *J* = 1H, 9.2 Hz), 5.16 (s, 1H), 4.75 (s, 1H), 3.91 (d, 2H, 6.0 Hz) 3.70 (s, 6H), 3.11 (t, 1H, 6.4 Hz, 6.4 Hz), 2.93 (t, 1H, 2 Hz, 6.0 Hz), 0.98 (s, 1H). ^13^C NMR (100 MHz, DMSO-d_6_) 167.0, 160.5, 157.9, 157.8, 152.8, 145.4, 144.4, 144.9, 143.0, 141.8, 135.8, 135.7, 129.6, 129.6, 127.6, 126.4, 126.3, 121.8, 120.3, 120.1, 120.0, 120.0, 113.0, 112.9, 111.9, 111.7, 109.9, 96.4, 85.2, 79.1, 64.8, 62.9, 54.9, 54.1, 45.7, 20.3. ESI-FT-ICR MS(HRMS) calcd for [M + H]^+^: 705.2571, found: 705.2568.

#### Compound 6

5 (0187 mg, 0.28 mmol), 0.25 M solution of 5-benzylthio-1H-tetrazole (2.48 mL) in dry CH_2_Cl_2_ (10 mL) and 2-cyanoethyl *N*,*N*,*N*′,*N*′-tetraisopropylphosphordiamidite (540 μL, 0.62 mmol) were mixed under N_2_ for 3 h. The crude mixture was dissolved in ethyl acetate. The solution containing 6 was washed with water, NaHCO_3_ aq. soln and brine. The organic layer was dried over MgSO_4_, and then filtered and the ethyl acetate was removed. The yellow foam (0.21 g, 0.24 mmol, 80%) was obtained and immediately used for DNA synthesis without further purification.

### Synthesis of oligodeoxynucleotide

The oligonucleotides having ^PCX^D were synthesized by a 3400 DNA synthesizer (Applied Biosystems) and purified by a HPLC system (JASCO PU-980, HG-980-31, DG-980-50, UV-970) with an InertSustainTM C18 column (GL Science, 5 μm, 10 × 150 mm). Synthesized oligonucleotides was identified by MALDI-TOF-MS.

### Photoirradiation and UPLC analysis

Photoirradiation was performed with an LED lamp (OminiCure, 400 nm, 8600 mW cm^−2^) on thermal cycler. The photoirradiated samples were analyzed with a UPLC system (Aquity, waters) equipped with BEH Shield RP18 column (1.7 μm, 2.1 × 50 mm, elution was with 0.05 M ammonium formate containing 1–10% CH_3_CN, linear gradient (10 min) at a flow rate of 0.4 mL min^−1^, 60 °C).

### Thermodynamic analysis of the hybridization

Thermodynamic parameters were obtained according to a method in the literature: where TM is the melting temperature of duplex, Δ*S*° is entropy and Δ*H* is the entropy of duplex formation, respectively. *R* is the gas constant and CT is the total strand concentration. TM was measured at various concentrations of duplex in 50 mM Na-cacodylate buffer (pH 7.4) containing 100 mM NaCl by a spectrophotometer (V-630bio, Jasco) equipped with a temperature controller.

## Conclusions

The ^PCX^D in double-stranded DNA photo-cross-linked to the pyrimidine base at the −1 position of the complementary strand was similar to that for ^PC^X, and the photoreactivity of ^PCX^D was significantly higher than that of ^PC^X. The photo-cross-linking rate of ^PCX^D having d-threoninol and cytosine was faster than that of ^PC^X and cytosine, the applicability of the photo-cross-linking reaction for DNA manipulation may be expanded by the use of ^PCX^D. We have already reported the importance of photo-cross-linking rate in photochemical antisense method in living cell and RNA FISH to *E. coli* 16S rRNA with a secondary structure. Therefore, this novel photo-cross-linker would contribute to the photoregulation of gene expression or biological events in living cells.

## Conflicts of interest

There are no conflicts to declare.

## Supplementary Material

RA-009-C9RA06145B-s001

## References

[cit1] Ziemianowicz D. S., Ng D., Schryvers A. B., Schriemer D. C. (2019). J. Proteome Res..

[cit2] Fu G., Dai Z. (2012). Talanta.

[cit3] Felczak M. M., Sage J. M., Huper-Kocurek K., Aykui S., Kaguni J. M. (2016). J. Biol. Chem..

[cit4] Goodsell D. S. (2001). Oncologist.

[cit5] Gasparro F. P., Havre P. A., Olack G. A., Gunther E. J., Glazer P. M. (1994). Nucleic Acids Res..

[cit6] Higuchi M., Kobori A., Yamayoshi A., Murakami A. (2009). Bioorg. Med. Chem..

[cit7] Rajendran A., Endo M., Katsuda Y., Hidaka K., Sugiyama H. (2011). J. Am. Chem. Soc..

[cit8] Churchil C. D., Eriksson L. A., Wetmore S. D. (2016). *J. Phys. Chem*. B.

[cit9] Nakatani K., Yoshida T., Saito I. (2002). J. Am. Chem. Soc..

[cit10] Sun H., Fan H., Eom H., Peng X. (2016). ChemBioChem.

[cit11] Fujimoto K., Sasago S., Mihara J., Nakamura S. (2018). Org. Lett..

[cit12] Fujimoto K., Yung H., Nakamura S. (2019). Chem.–Asian J..

[cit13] Fujimoto K., Hashimoto M., Watanabe N., Nakamura S. (2019). Bioorg. Med. Chem. Lett..

[cit14] Asanuma H., Takarada T., Yoshida T., Tamura D., Liang X., Komiyama M. (2001). Angew. Chem., Int. Ed..

[cit15] Murayama K., Tanaka Y., Toda T., Kashida H., Asanuma H. (2013). Chemistry.

[cit16] Sakamoto T., Tanaka Y., Fujimoto K. (2015). Org. Lett..

[cit17] Beaucage S. L., Caruthers M. H. (1981). Tetrahedron Lett..

[cit18] Mergny J. L., Lacroix L. (2003). Oligonucleotides.

[cit19] OECD Guideline for testing of chemicals 117, http://www.oecd.org/chemicalsafety/risk-assessment/1948177.pdf

