# Supplementary material for: DNA photo-cross-linking using a pyranocarbazole-modified oligodeoxynucleotide with a d-threoninol linker
Source: RSC Adv. 2019 Sep 27;9(53):30693–7. doi: 10.1039/c9ra06145b (PMC9072208; doi:10.1039/c9ra06145b)
Supplement: RA-009-C9RA06145B-s001 [file RA-009-C9RA06145B-s001.pdf]

Electronic Supplementary Material (ESI) for RSC Advances.
This journal is © The Royal Society of Chemistry 2019

Supporting Information

DNA photo-cross-linking using pyranocarbazole modified oligodeoxynucleotide with D-threoninol linker

Kenzo Fujimoto*[a], Tsubasa Yamaguchi[a], Takahiro Inatsugi[b], Masahiko Takamura[b], Isao Ishimaru[b], Ayako Koto[b], Shigetaka Nakamura[a]

Compound 2

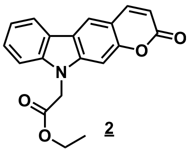

2

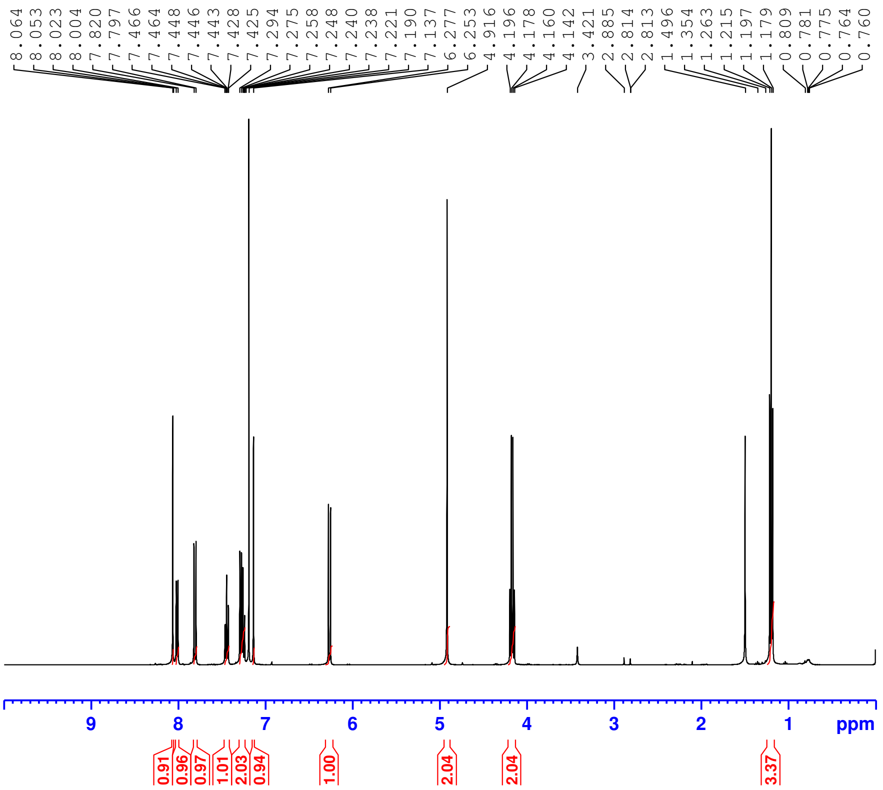

Figure S1. 1H-NMR spectra of Compound 2

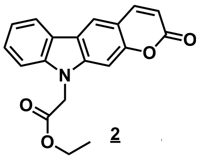

2

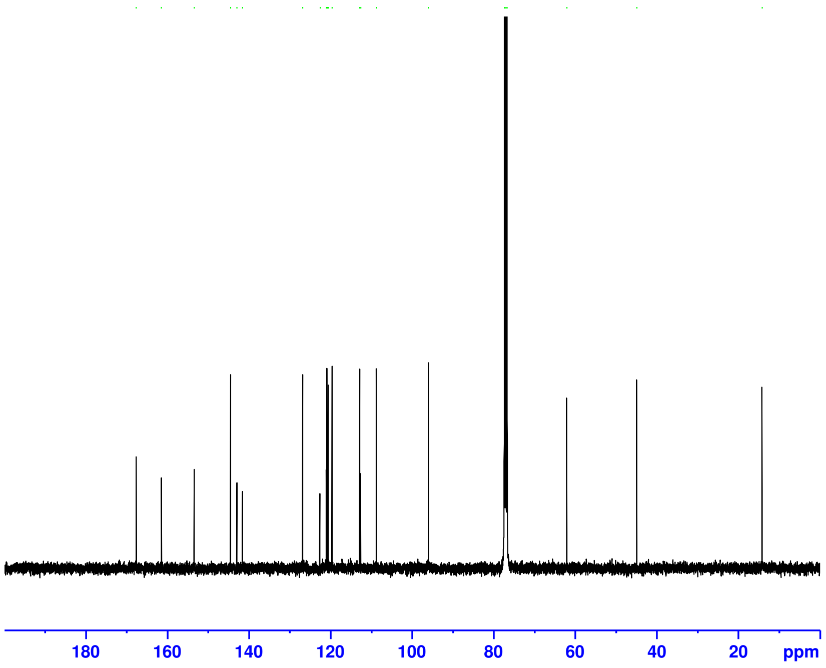

Figure S2. 13C-NMR spectra of Compound 2

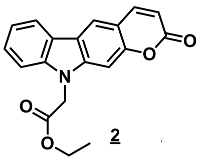

2

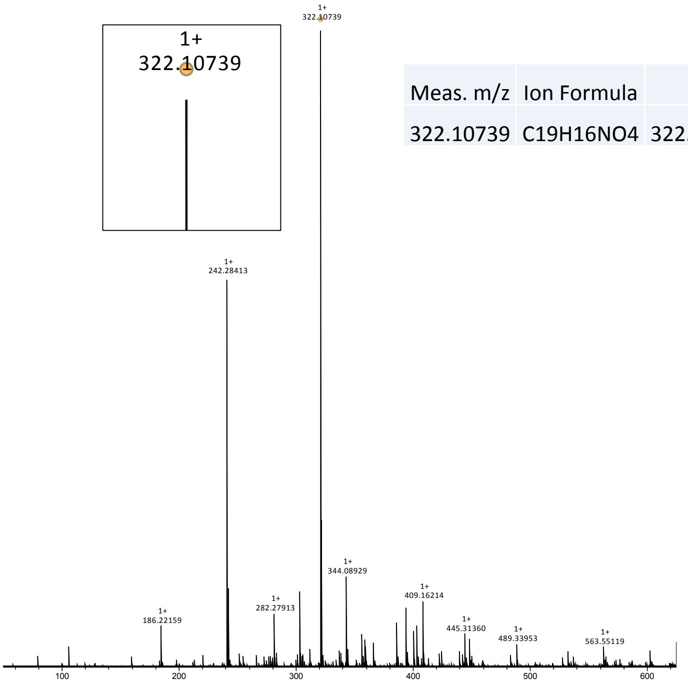

Meas. m/z	Ion Formula	m/z	err [ppm]
322.10739	C19H16NO4	322.107384	0

Figure S3. ESI analysis of Compound 2

Compound 3

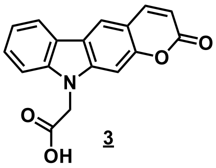

3

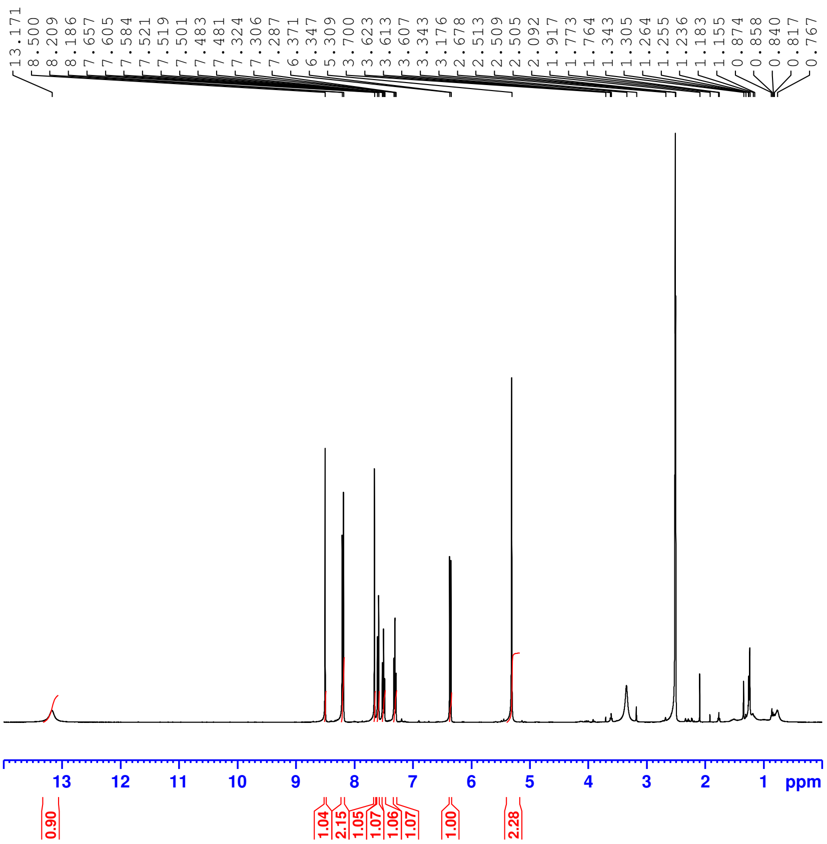

Figure S4. 1H-NMR spectra of Compound 3

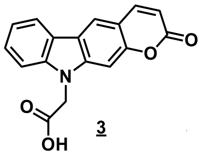

3

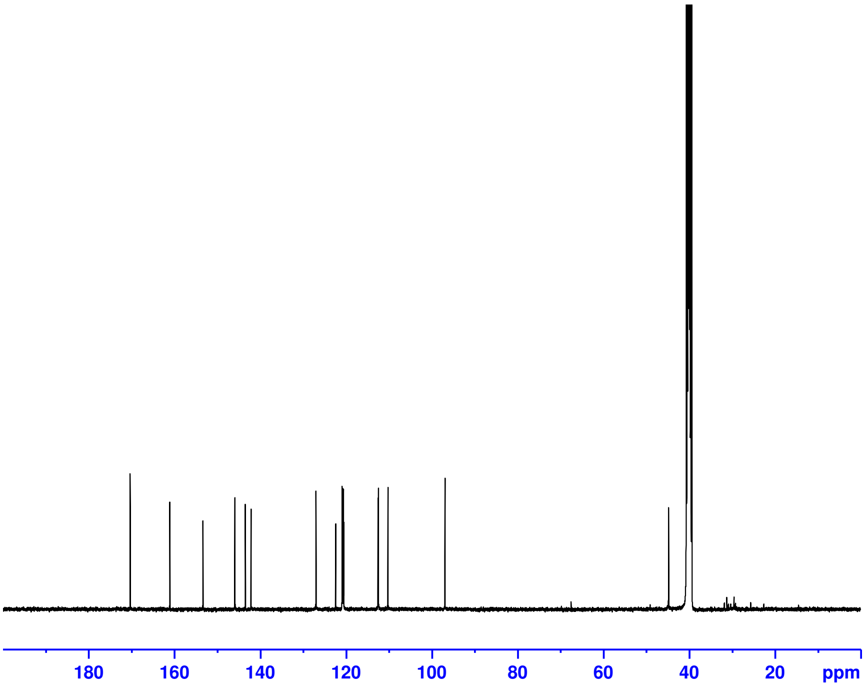

Figure S5. 13C-NMR spectra of Compound 3

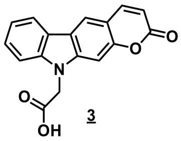

3

Meas. m/z	Ion Formula	m/z	err [mDa]
294.076204	C17H12NO4	294.076084	-0.1
316.058122	C17H11NNaO4	316.058029	-0.1

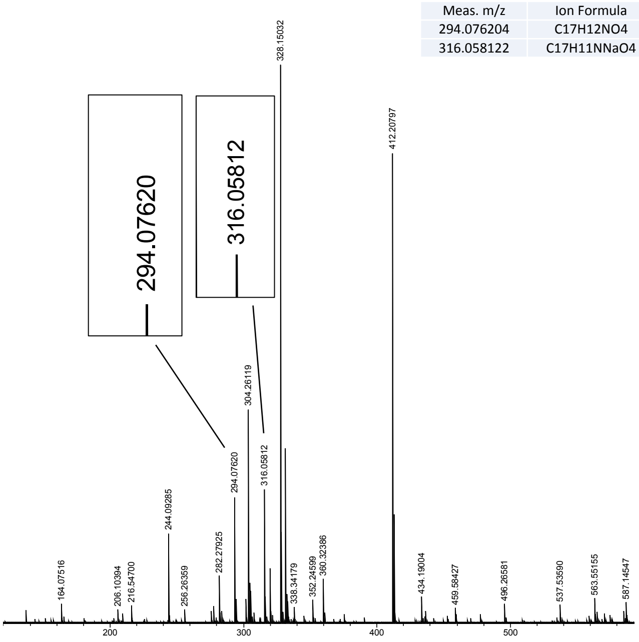

Figure S6. ESI analysis of Compound 3

Compound 4

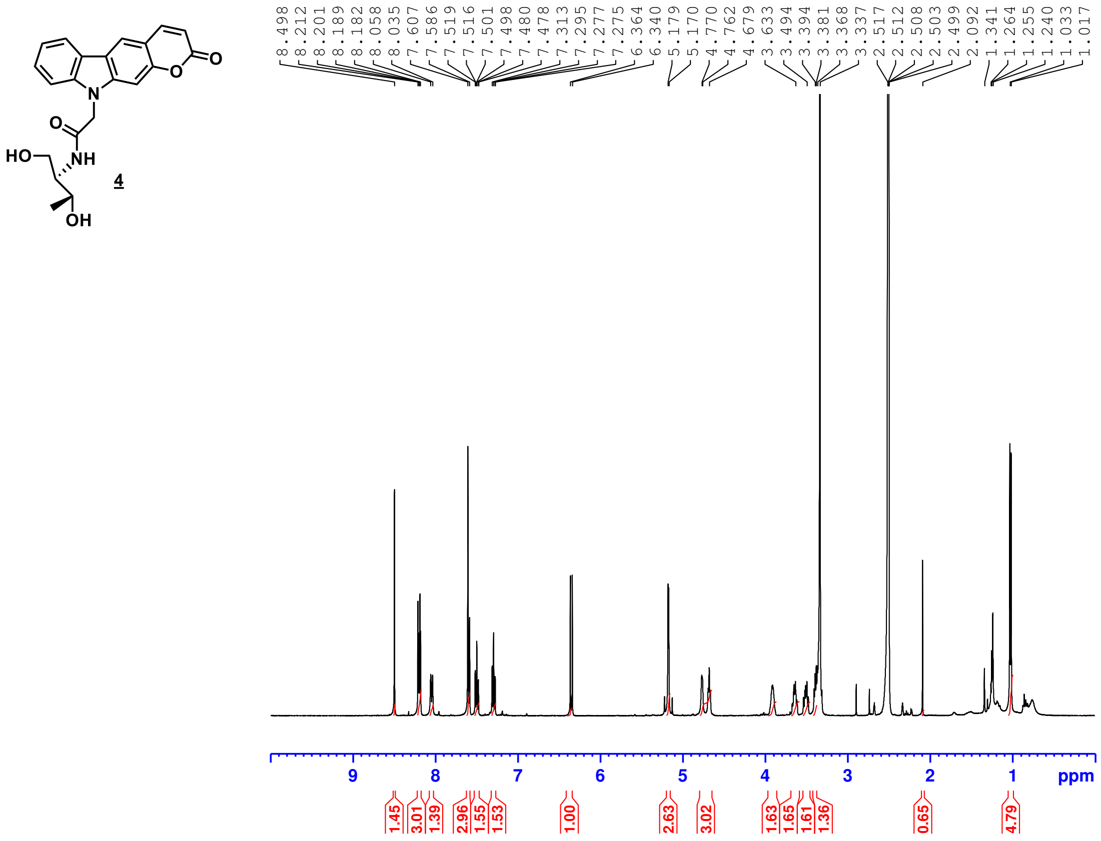

Figure S7. 1H-NMR spectra of Compound 4

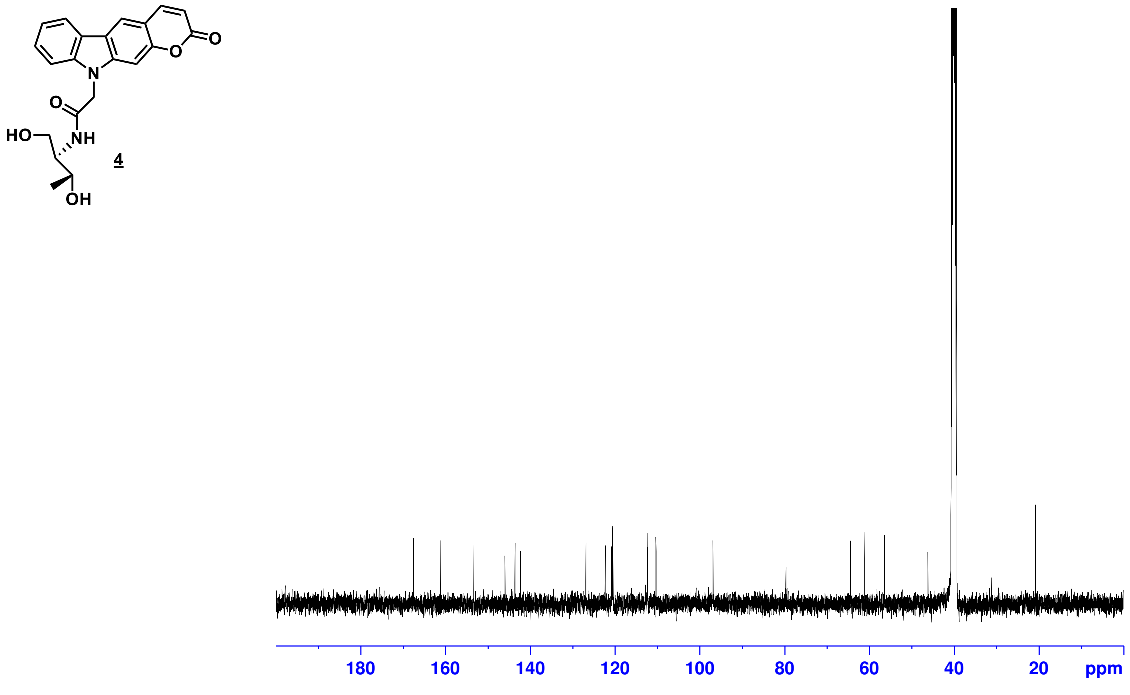

Figure S8. 13C-NMR spectra of Compound 4

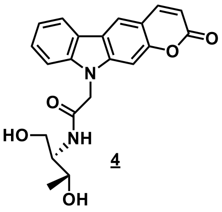

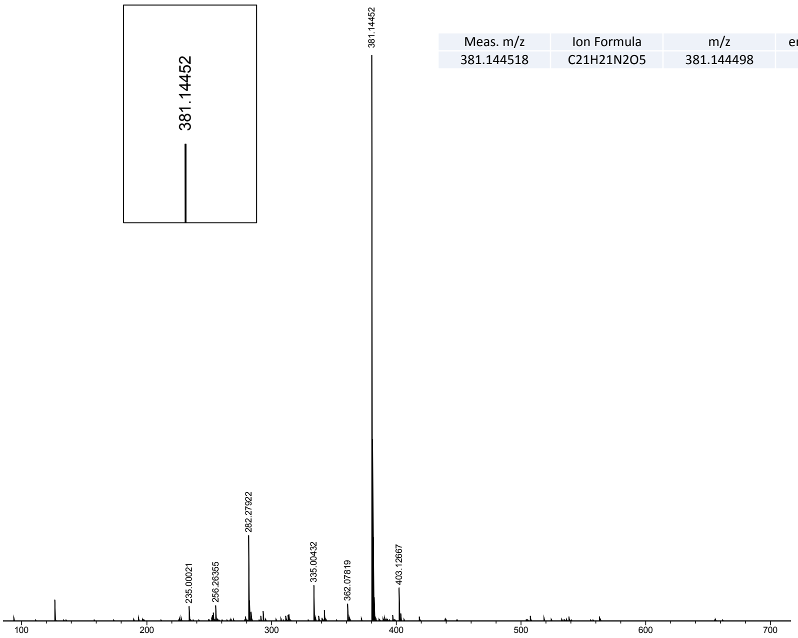

Meas. m/z	Ion Formula	m/z	err [mDa]
381.144518	C21H21N2O5	381.144498	0

Figure S9. ESI analysis of Compound 4

Compound 5

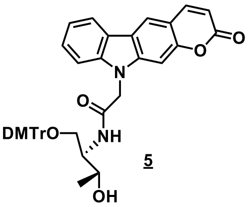

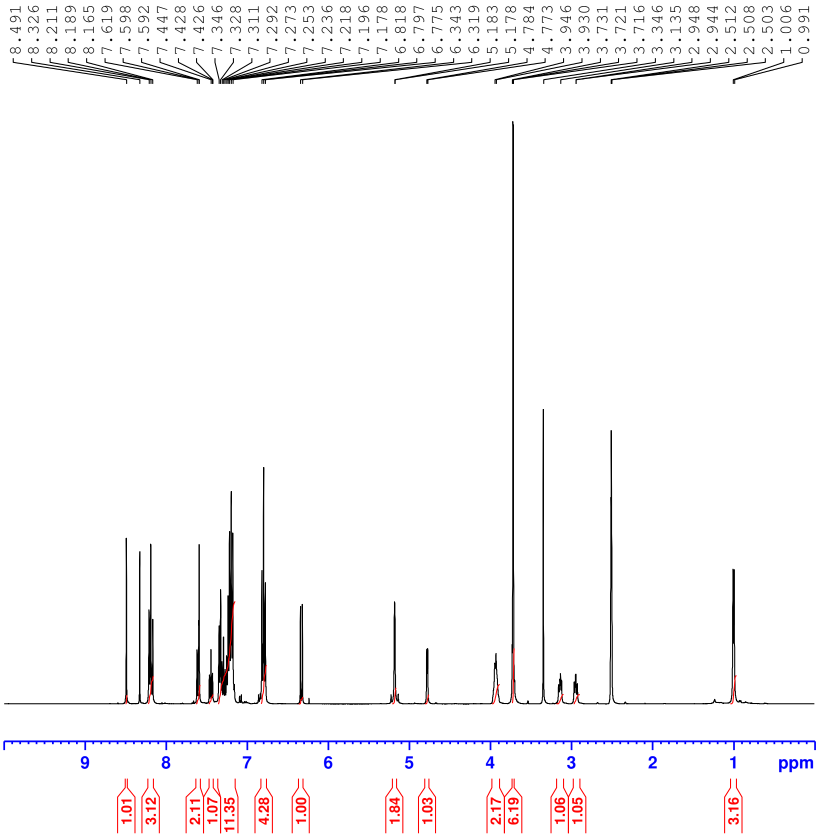

Figure S10. 1H-NMR spectra of Compound 5

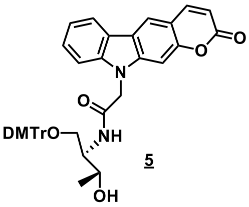

5

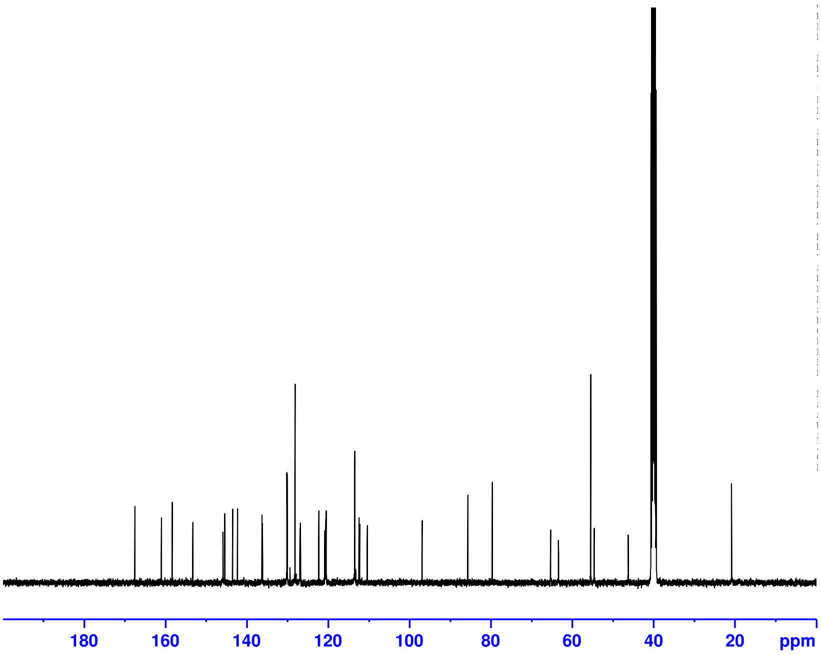

Figure S11. 13C-NMR spectra of Compound 5

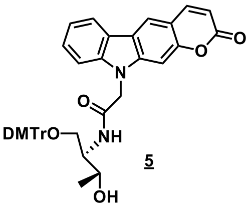

5

Meas. m/z	Ion Formula	m/z	err [ppm]
705.256833	C42H38N2NaO7	705.257122	0.4

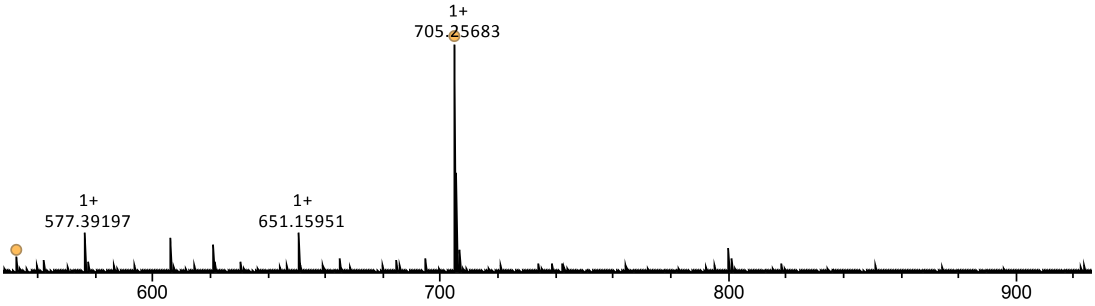

Figure S12. ESI analysis of Compound 5

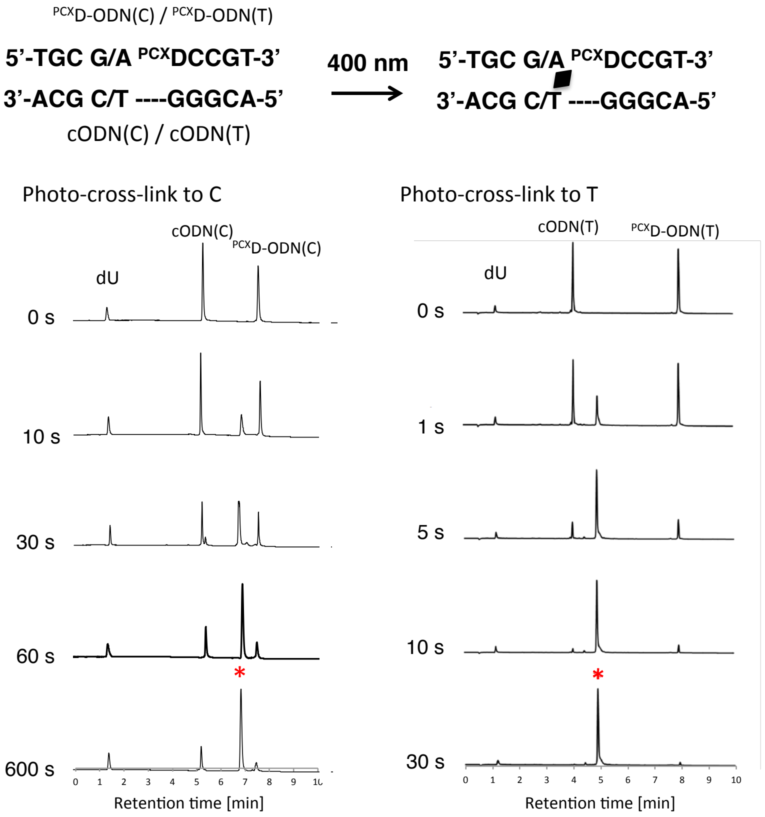

Figure S13 Photo-cross-linking using ^{PCX}D. 10 μM ODNs in 50 mM Cacodylate buffer containing 100 mM NaCl was annealed and photoirradaition at 400 nm on ice.

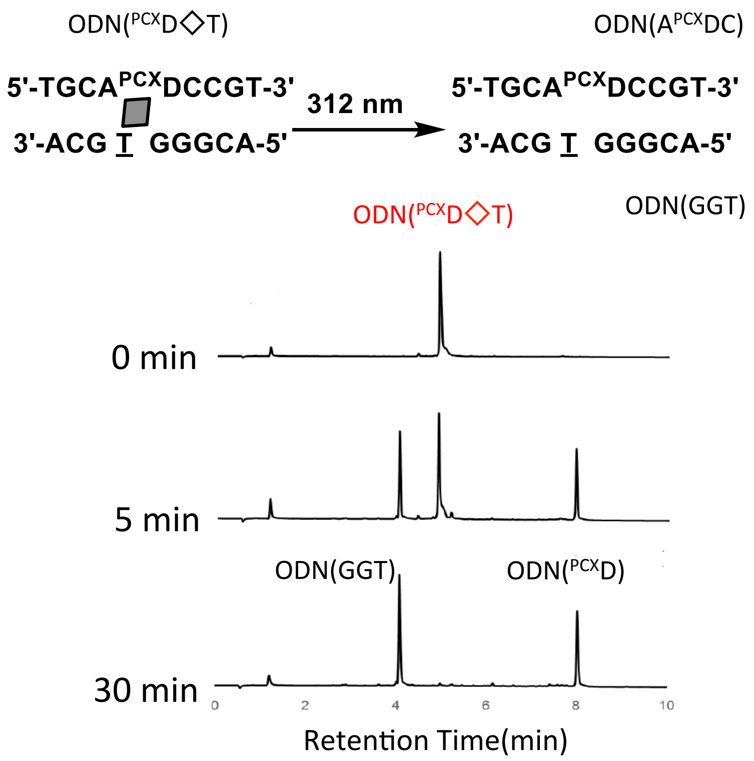

Figure S14 The photo-splitting of ^{PCX}D. The 10 μM photoadduct in 50 mM Cacodylate buffer containing 100 mM NaCl was photoirradaited at 312 nm under the 60°C condition.

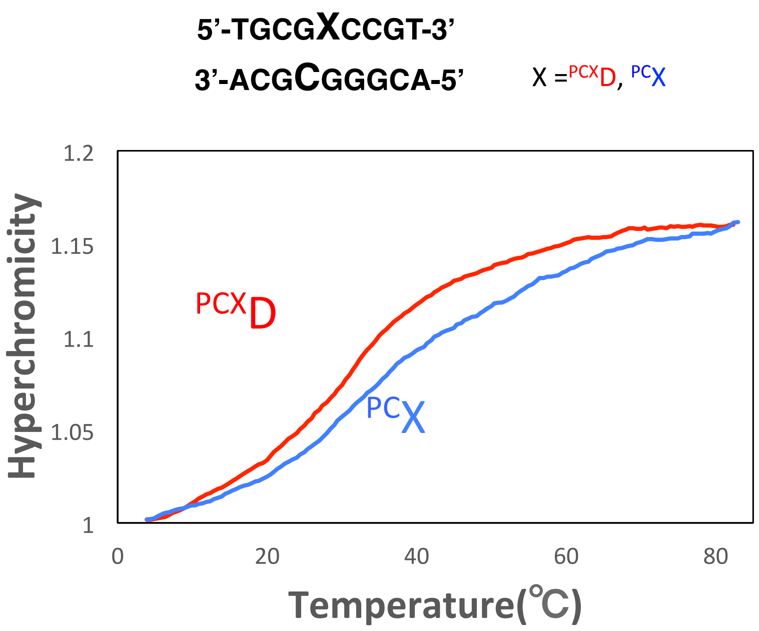

Figure S15 Meitng Curve of duplex cnontaining. Sample solution is duplex in 50 mM Cacodylate buffer containing 100 mM NaCl was measured absorbance at 260 nm from 5°C to 85°C

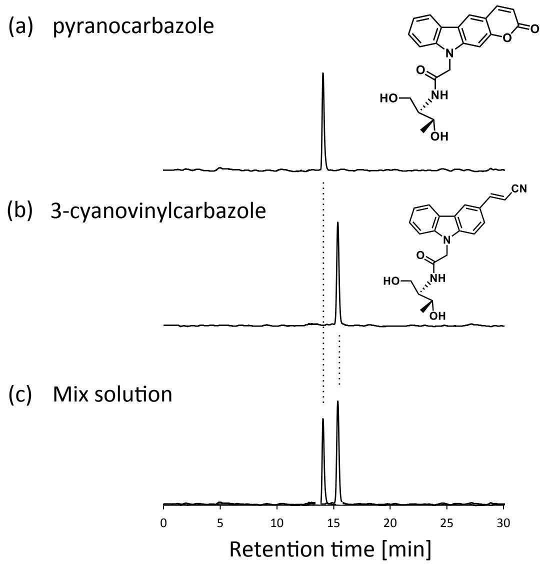

	Retention time [min]	LogP
^{PCX}D	14.1	1.12
^{CNV}D	15.2	1.35

Figure S16 The HPLC analysis of (a) pyranocarbazole (b) 3-cyanovinylcarbazole, and (c) Both

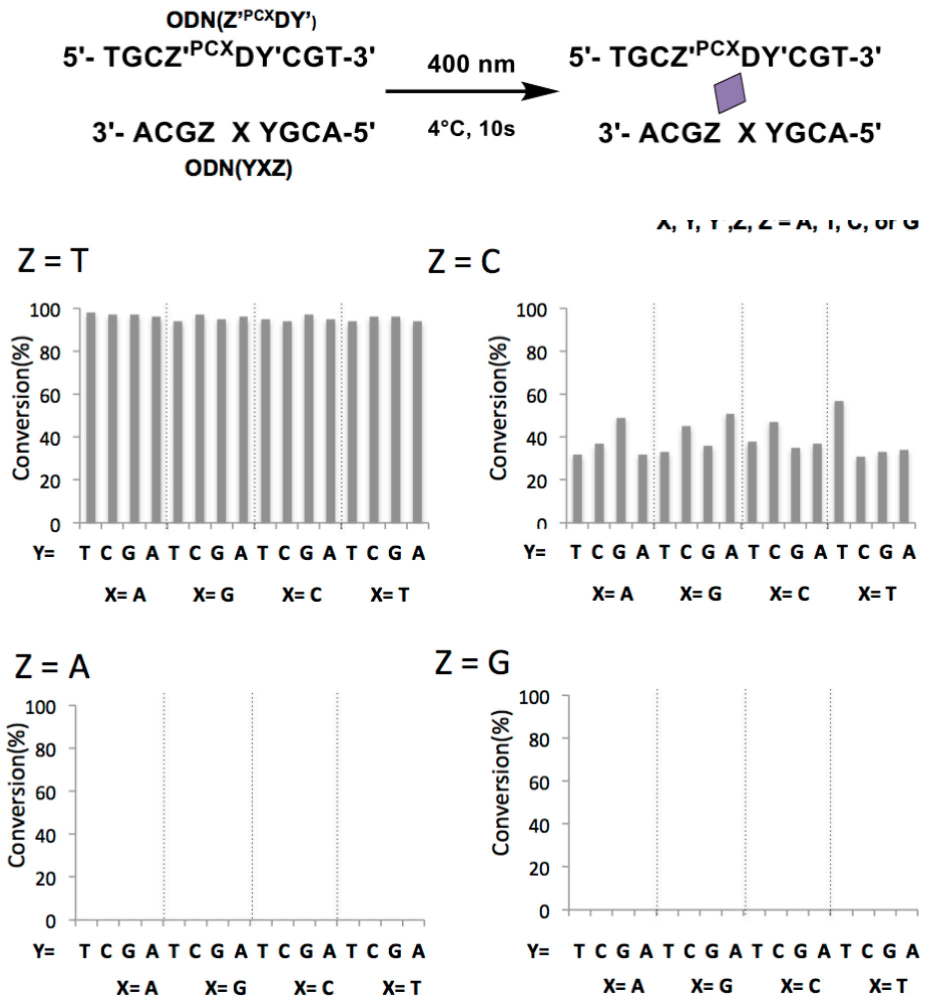

Figure. S17 Effect of surrounding base of ^{PCX}D on its photoreactivity in dsDNA. 10 μM DNA in 50 mM cacodylate buffer (pH 7.4) containing 100 mM NaCl was 400 nm-irradiated for 10 s at 4 °C.

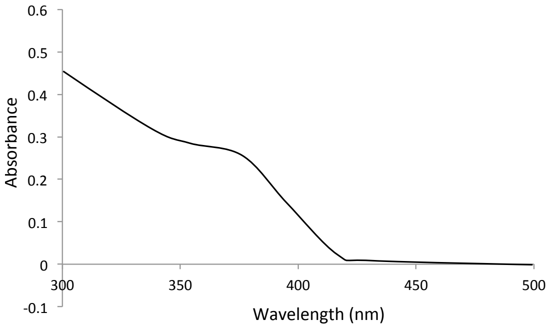

Figure S18 UV spectra of ^{PCX}D

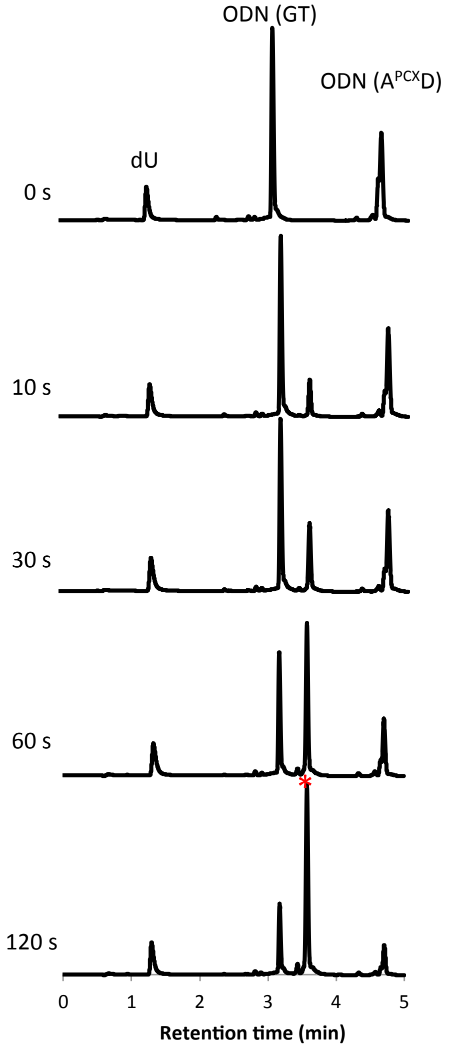

Figure S19 Photo-cross-linking using ^{PCX}D. 10 μM ODNs in 50 mM Cacodylate buffer containing 100 mM NaCl was annealed and photoirradaition at 450 nm on ice.
